# Undertreatment of Anticoagulant Therapy in Hospitalized Acute Ischemic Stroke Patients With Atrial Fibrillation

**DOI:** 10.3389/fcvm.2022.841020

**Published:** 2022-03-30

**Authors:** Xiaoxian Gong, Hongfang Chen, Jianan Wang, Wansi Zhong, Luowei Chen, Shenqiang Yan, Min Lou

**Affiliations:** ^1^Department of Neurology, The Second Affiliated Hospital of Zhejiang University, School of Medicine, Hangzhou, China; ^2^Department of Neurology, Jinhua Hospital of Zhejiang University, Jinhua Municipal Central Hospital, Jinhua, China

**Keywords:** acute ischemic stroke, atrial fibrillation, anticoagulant therapy, cerebral infarction, undertreatment

## Abstract

**Background:**

This study aimed to investigate the prevalence and factors associated with the initiation of oral anticoagulation among patients with acute ischemic stroke (AIS) and concurrent atrial fibrillation (AF) at discharge in China.

**Methods:**

We continuously included hospitalized patients with AIS with an AF diagnosis registered in the computer-based Online Database of Acute Stroke Patients for Stroke Management Quality Evaluation (CASE II) from January 2016 to December 2020 and divided them into a and non-anticoagulant groups according to the medications at discharge. Binary logistic regression was used to determine the factors associated with the prescription of anticoagulants in patients with AF.

**Results:**

A total of 16,162 patients were enrolled. The mean age was 77 ± 9 years, 8,596 (53.2%) were males, and the median baseline National Institute of Health Stroke Scale score was 5 (2–12). Of the 14,838 patients without contraindications of antithrombotic therapy, 6,335 (42.7%) patients were initiated with anticoagulation treatment at discharge. Prior history of hemorrhagic stroke (OR 0.647, *p* < 0.001) and gastrointestinal bleeding (OR 0.607, *p* = 0.003) were associated with a lower rate of anticoagulation at discharge. Patients with any intracranial hemorrhage (OR 0.268, *p* < 0.001), gastrointestinal bleeding (OR 0.353, *p* < 0.001), or pneumonia during hospitalization (OR 0.601, *p* < 0.001) were less likely to receive anticoagulants at discharge. Among 7,807 patients with previously diagnosed AF and high risk of stroke (CHA_2_DS_2_-VASc ≥2), only 1,585 (20.3%) had been receiving anticoagulation treatment prior to the onset of stroke. However, the mean international normalized ratio (INR) was 1.5 on the first test during hospitalization in patients receiving warfarin. Patients complicated with a previous history of ischemic stroke/transient ischemic attack (TIA; OR 2.303, *p* < 0.001) and peripheral artery disease (OR 1.456, *p* = 0.003) were more common to start anticoagulants.

**Conclusions:**

Less than half of patients with AIS and concurrent AF initiated guideline-recommended oral anticoagulation at discharge, while only 20% of patients with previously diagnosed AF with a high risk of stroke had been using anticoagulants prior to the onset of stroke, which highlights a large care gap in hospitalized stroke patients and the importance of AF management.

## Introduction

Atrial fibrillation (AF) increases the risk of ischemic stroke from thromboembolism, and oral anticoagulation (OAC) is recommended for preventing ischemic strokes (1, 2). Patients who receive OAC according to guideline recommendations have a better prognosis compared with those whose treatment deviated from guideline recommendations. Meanwhile, a lower rate of OAC was found to be associated with increased incidence of ischemic stroke and all-cause mortality (3). However, AF undertreatment is still a long-established healthcare concern. Multiple studies investigating the prevalence of AF undertreatment among patients with stroke have reported that the proportion of patients who did not receive OAC treatment varied worldwide, ranging from approximately 20 to 60%, which highlighted a large care gap and an opportunity to improve AF management (3–7). In Asia, the proportion of insufficient treatment is generally higher (6, 8, 9), but there is still a lack of large data about the undertreatment of anticoagulant therapy in hospitalized patients with acute ischemic stroke (AIS) with AF. Moreover, it remains unclear what factors affect an individual's risk of undertreatment. Thus, this study aimed to investigate the point prevalence and factors associated with the initiation of anticoagulation among hospitalized patients with AIS and concurrent AF in China.

## Methods

### Data Collection and Monitoring

This study came from the Computer-based Online Database of Acute Stroke Patients for Stroke Management Quality Evaluation (CASE-II, NCT04487340), a multicenter prospective registry. Initiated in 2016, CASE-II was designed to examine the current status of stroke care in China to help develop strategies to improve stroke care. The medical documents during hospitalization of consecutive patients with stroke were collected through a special electronic data capture system. Briefly, original hospital records of patients were saved as images or portable document formats. Specific software pre-processed the above materials and sent them to multiple Optical Character Recognition (10) engines to build documents with recognized text, which were subsequently re-segmented and synthesized. Required data was extracted from the post-processed text, and the cross-check of each case was carried out by a quality control team. Only the de-identified documents were preserved in a safe information database and monitored by an independent contract research organization throughout the study period.

### Patients

The CASE-II has consecutively recruited patients with stroke who were diagnosed with AIS, transient ischemic attack (TIA), hemorrhagic stroke, or subarachnoid hemorrhage and admitted within 7 days of symptom onset in China. Because patient information in the CASE-II was de-identified and anonymized before being released to the researchers, the informed consent requirement was waived by Institutional Review Board. For the present analysis, patients with AIS with concurrent AF aged ≥18 years were enrolled in the CASE-II between January 2016 and December 2020. AIS was diagnosed according to the World Health Organization criteria (11) and confirmed by computed tomography or magnetic resonance imaging. AF was defined as atrial arrhythmia with irregular R-R intervals and no clear repetitive P waves, and diagnosed with an electrocardiograph, 24- or 48-h Holter, or telemonitoring with recording and automated rhythm detection. Patients with incomplete information on anticoagulation at discharge or who died during hospitalization were excluded.

### Variables

We collected the following patient information during hospitalization: demographics (age, sex); blood pressure at admission, baseline National Institutes of Health Stroke Scale (NIHSS) score, and baseline CHA_2_DS_2_-VASc score; medical history and medication history; reperfusion therapy (intravenous thrombosis with recombinant tissue-type plasminogen activator, mechanical thrombectomy with stent retrievers, and/or thromboaspiration, balloon angioplasty, stenting, or intra-arterial thrombolysis); laboratory tests at admission; medications usage at discharge; and reasons for non-treatment were documented in patient records, which include medical contraindications, refusal against medical advice, or transfer to another hospital.

### Statistical Analysis

Clinical characteristics were summarized by computing the mean [standard deviation (SD)] or median [interquartile range (IQR)]. Differences between the two groups were estimated by the *t*-test or Mann-Whitney U test if they were continuous variables. Categorical variables were summarized by proportion (n), and differences between the two groups were estimated by the Pearson χ^2^ test. To avoid overfitting, we ran logistic regression with a lasso regularization to select variables associated with the use of anticoagulation. Lambda parameters, which minimize the 10-fold cross-validation prediction error rate, were determined automatically using the function cv. glmnet (12). Function fixed Lasso Inf was used to compute *p*-values and confidence intervals for the lasso estimate. When analyzing the factors associated with prior use of anticoagulation in previously diagnosed patients with AF with a high risk of stroke, clinical information prior to the index stroke was included in the multivariate analysis. All comparisons were two-sided, with statistical significance defined as *p* < 0.05. All statistical analysis was performed using SPSS, Version 24.0 (IBM, Armonk, New York), and R software, Version 4.1.0 (R Foundation, Vienna, Austria).

## Results

### Patient Characteristics of the Study Population

**Figure 1:**
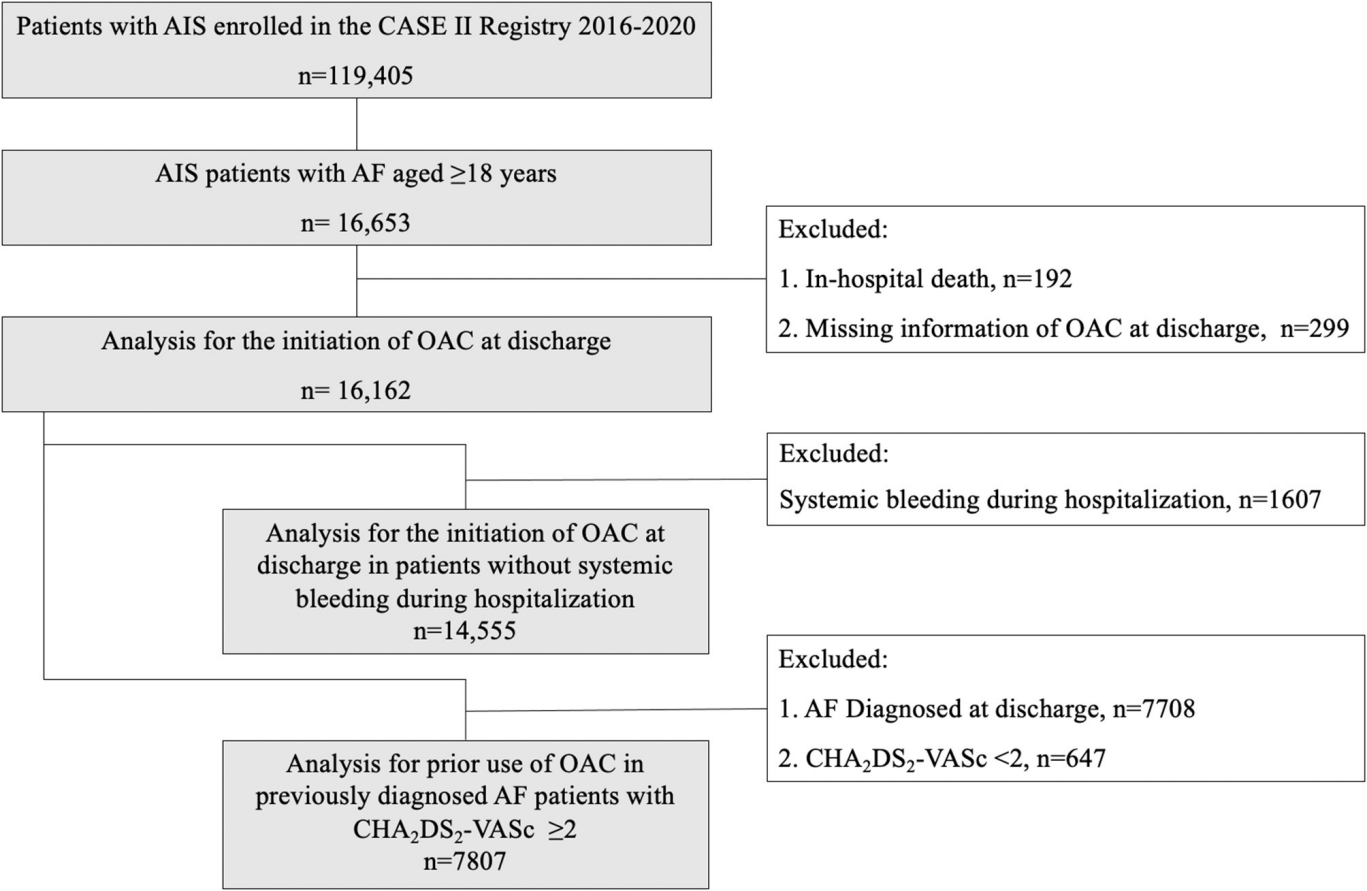
Flow chart of study population identification. AIS indicates acute ischemic stroke; AF, atrial fibrillation; CASE II, Computer-based Online Database of Acute Stroke Patients for Stroke Management Quality Evaluation; OAC, oral anticoagulation.

**Figure 2:**
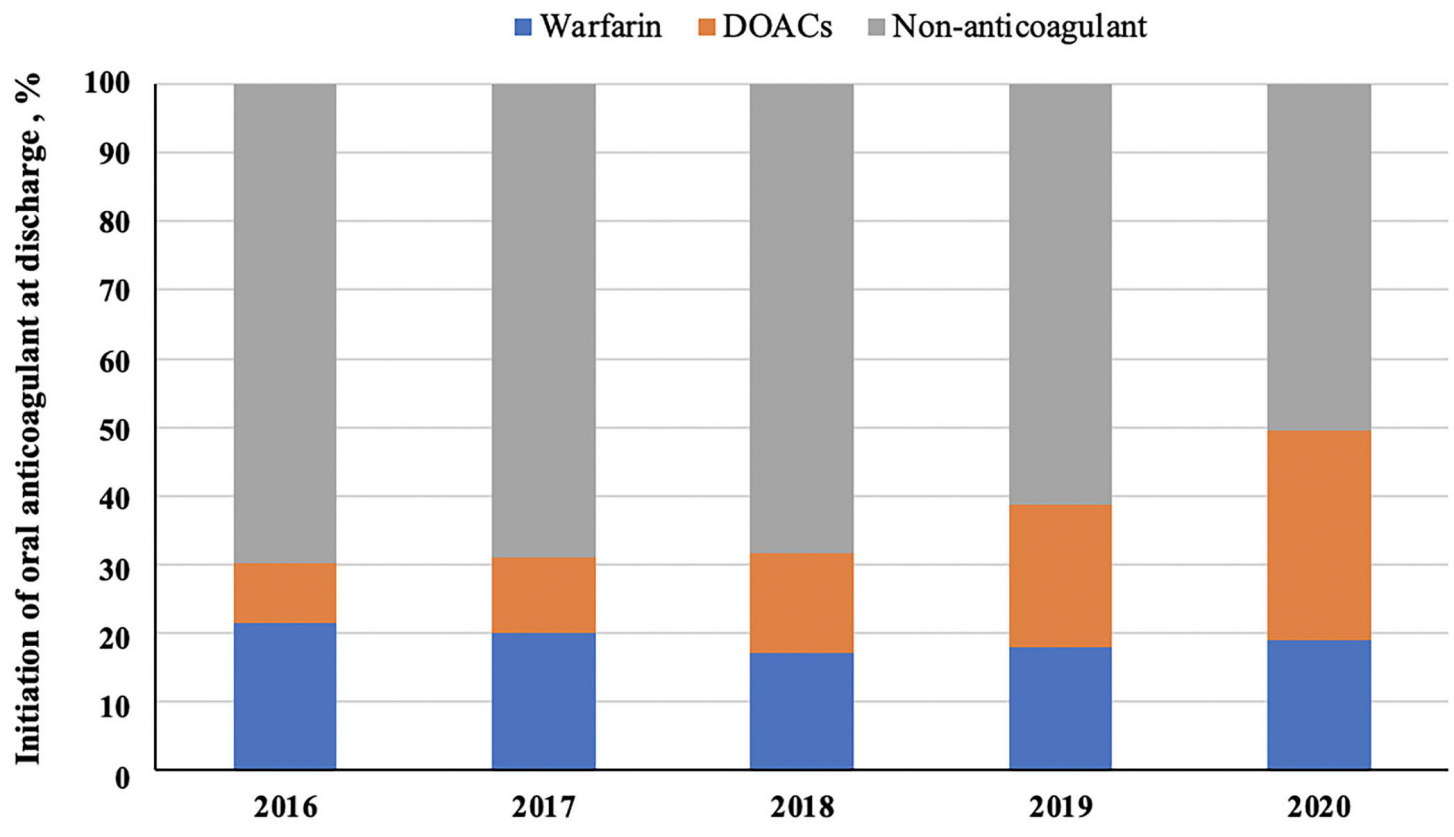
The trends of oral anticoagulant use at discharge. DOACs indicate direct oral anticoagulants.

A total of 16,162 patients with AIS and concurrent AF were included for analysis in this study (Figure 1). Mean age was 77 ± 9 years, 8,596 (53.2%) were males, and median baseline NIHSS score was 5 (2–12). There were 14,838 patients having no clear documentation of antithrombotic contraindications at discharge. Among them, 5,895 patients (39.7%) received antiplatelets, 6,061 (40.8%) were on OAC, 274 (1.8%) were on both antiplatelet and OAC, while 2,608 patients (17.6%) did not receive any antithrombotic agents (Table 1). Initiation of anticoagulation at discharge was increased from 30.1% (52 of 173) in 2016 to 49.6% (2,266 of 4,573) in 2020. From Figure 2, we could observe an increased use of direct oral anticoagulants (DOACs) [from 8.7% (15 of 173) to 30.5% (1,397 of 4,573)] and a slight decline of warfarin usage [from 21.4% (37 of 173) to 19.0% (869 of 4573)].

**Table 1:** Characteristics of acute ischemic stroke patients with atrial fibrillation.

**Variable**	
Number of patients	16,162
Age, years, Mean ± SD	77 ± 9
Male, n (%)	8,596 (53.2)
Baseline NIHSS score, (median [IQR])^*^	5 (2–12)
CHA_2_DS_2_-VASc score, (median [IQR])	4 (3–5)
**Prior medical history**	
Ischemic stroke/TIA, n (%)	4,544 (28.1)
Hypertension, n (%)	13,320 (82.4)
Diabetes, n (%)	3,118 (19.3)
Dyslipidemia, n (%)	5,796 (35.9)
Coronary heart disease, n (%)	3,190 (19.7)
Smoking, n (%)	3,965 (24.5)
Reperfusion therapy, n (%)	2,919 (18.1)
**Number of eligible patients for antithrombotic agents at discharge**	14,838
Antiplatelets only, n (%)	5,895 (39.7)
Anticoagulants only, n (%)	6,061 (40.8)
Antiplatelets and Anticoagulants, n (%)	274 (1.8)
No-antithrombotic agent due to clear documentation of refusal or transfer to another hospital, n (%)	904 (6.1)
No-antithrombotic agent due to unknown reasons, n (%)	1,704 (11.5)
**Types of anticoagulants**	6,335
Warfarin, n (%)	2,916 (46.0)
Direct oral anticoagulants, n (%)	3,419 (54.0)

*IQR indicates interquartile range; SD, standard deviation; NIHSS, National Institutes of Health Stroke Scale; TIA, Transient ischemic attack*.

* *NIHSS was available for 13,766 patients*.

### Factors Associated With the Initiation of Anticoagulation in AIS Patients With AF at Discharge

As shown in Table 2, patients with the initiation of anticoagulants at discharge were younger (OR 0.973, 95% CI 0.970–0.977), had lower systolic blood pressure (OR 0.998, 95% CI 0.996–0.999), and baseline NIHSS score (OR 0.943, 95% CI 0.937–0.947) than patients who were not on OAC treatment. The prior anticoagulants usage (OR 3.408, 95% CI 3.065–3.789), complication with deep vein thrombosis (OR 1.711, 95% CI 1.446–2.020), and longer hospital stay (OR 1.044, 95% CI 1.039–1.050) were all associated with increased odds of receiving anticoagulation. The previous medical history associated with lower frequency of anticoagulation at discharge includes ischemic stroke/TIA (OR 0.890, 95% CI 0.826–0.965), hemorrhagic stroke (OR 0.647, 95% CI 0.527–0.795), and gastrointestinal bleeding (OR 0.607, 95% CI 0.463–0.804). In addition, patients with any intracranial hemorrhage (OR 0.268, 95% CI 0.232–0.309), gastrointestinal bleeding (OR 0.353, 95% CI 0.263–0.475), pneumonia during hospitalization (OR 0.601, 95% CI 0.553–0.652), or combined with renal insufficiency (OR 0.841, 95% CI 0.752–0.952) were less likely to receive anticoagulants at discharge.

**Table 2:** Univariate and multivariate analysis for the initiation of anticoagulation at discharge.

**Variable**	**Univariate analysis**			**Multivariate analysis**		
	**Non-anticoagulant group**	**Anticoagulant group**	***P* value**	**OR**	**(95% CI)**	***P* value**
	**(*n* =9,827)**	**(*n* = 6,335)**				
Age, years, Mean ± SD	78 ± 9	75 ± 10	<0.001	0.973	(0.970–0.977)	<0.001
Male, n (%)	5,061 (51.5)	3,535 (55.8)	<0.001	1.014	(0.526–1.120)	0.786
**Prior medical history**						
Ischemic stroke/TIA, n (%)	2,825 (28.7)	1,719 (27.1)	0.026	0.890	(0.826–0.965)	0.011
Hemorrhagic stroke, n (%)	327 (3.3)	125 (2.0)	<0.001	0.647	(0.527–0.795)	<0.001
Gastrointestinal bleeding, n (%)	187 (1.9)	73 (1.2)	<0.001	0.607	(0.463–0.804)	0.003
Urinary tract bleeding, n (%)	3 (0)	2 (0)	0.971	-		
Smoking, n (%)	2,271 (23.1)	1,694 (26.7)	<0.001	1.029	(0.760–1.471)	0.400
Anticoagulants, n (%)	585 (6.0)	1,173 (18.5)	<0.001	3.408	(3.065–3.789)	<0.001
**New comorbidity during hospitalization**						
Any intracranial bleeding, n (%)	1,112 (11.3)	222 (3.5)	<0.001	0.268	(0.232–0.309)	<0.001
Gastrointestinal bleeding, n (%)	427 (4.3)	122 (1.9)	<0.001	0.353	(0.263–0.475)	<0.001
Urinary tract bleeding, n (%)	14 (0.1)	11 (0.2)	0.622	-		
Pneumonia, n (%)	3,345 (34.0)	1,113 (17.6)	<0.001	0.601	(0.553–0.652)	<0.001
Hypertension, n (%)	8,098 (82.4)	5,222 (82.4)	0.967	-		
Diabetes, n (%)	1,885 (19.2)	1,233 (19.5)	0.658	-		
Dyslipidemia, n (%)	3,401 (34.6)	2,395 (37.8)	<0.001	1.044	(0.910–1.112)	0.324
Coronary heart disease, n (%)	2,030 (20.7)	1,160 (18.3)	<0.001	0.942	(0.869–1.095)	0.258
Heart failure, n (%)	402 (4.1)	180 (2.8)	<0.001	0.884	(0.745–1.241)	0.282
Renal insufficiency, n (%)	961 (9.8)	498 (7.9)	<0.001	0.841	(0.752–0.952)	0.013
Peripheral artery disease, n (%)	404 (4.1)	297 (4.7)	0.079	-		
Anemia, n (%)	444 (4.5)	203 (3.2)	<0.001	0.823	(0.696–1.018)	0.062
Deep vein thrombosis, n (%)	308 (3.1)	252 (4.0)	0.004	1.711	(1.446–2.020)	<0.001
SBP, mmHg, Mean ± SD	151 ± 24	148 ± 23	<0.001	0.998	(0.996–0.999)	0.003
DBP, mmHg, Mean ± SD	85 ± 15	85 ± 15	0.98	-		
Baseline NIHSS score, Median (IQR)^*^	6 (2,14)	3 (1,7)	<0.001	0.943	(0.937–0.947)	<0.001
Reperfusion therapy, n (%)	1,786 (18.2)	1,133 (17.9)	0.64	-		
Length of stay, days, Median (IQR)	11 (8–15)	11 (9–15)	<0.001	1.044	(1.039–1.050)	<0.001

*CI indicates confidence interval; DBP, diastolic blood pressure; IQR, interquartile range; NIHSS, national institutes of health stroke scale; OR, odds ratio; SD, standard deviation; SBP, systolic blood pressure; TIA, transient ischemic attack*.

* *NIHSS was available for 13,766 patients (5,570 in anticoagulant group and 8,196 in non-anticoagulant group)*.

Additional sensitivity analyses were performed to determine the factors associated with anticoagulant therapy at discharge in patients without systemic bleeding during hospitalization, and the findings were generally comparable with that in the primary analysis (Table 3).

**Table 3:** Univariate and multivariate analysis for the initiation of anticoagulation at discharge in patients without systemic bleeding during hospitalization.

**Variable**	**Univariate analysis**			**Multivariate analysis**		
	**Non-anticoagulant group (*n* = 8,498)**	**Anticoagulant group (*n* = 6,057)**	***P* value**	**OR**	**(95% CI)**	***P* value**
Age, years, Mean ± SD	78 ± 9	75 ± 9	<0.001	0.980	(0.970–0.978)	<0.001
Male, n (%)	4,389 (51.6)	3,375 (55.7)	<0.001	1.011	(0.385–1.119)	0.832
**Prior medical history**						
Ischemic stroke/TIA, n (%)	2,488 (29.3)	1,638 (27.0)	0.003	0.875	(0.811–0.949)	0.005
Hemorrhagic stroke, n (%)	297 (3.5)	116 (1.9)	<0.001	0.582	(0.468–0.723)	<0.001
Gastrointestinal bleeding, n (%)	173 (2.0)	67 (1.1)	<0.001	0.546	(0.411–0.725)	<0.001
Urinary tract bleeding, n (%)	3 (0)	2 (0)	0.942	-		
Smoking, n (%)	1,949 (22.9)	1,608 (26.5)	<0.001	1.027	(0.723–1.733)	0.369
Anticoagulants, n (%)	482 (5.7)	1,123 (18.5)	<0.001	3.525	(3.152–3.939)	<0.001
**New comorbidity during hospitalization**						
Hypertension, n (%)	6,983 (82.2)	5,000 (82.5)	0.557	-		
Diabetes, n (%)	1,597 (18.8)	1,162 (19.2)	0.552	-		
Dyslipidemia, n (%)	2,982 (35.1)	2,284 (37.7)	0.001	1.026	(0.782–1.088)	0.611
Pneumonia, n (%)	2,685 (31.6)	1,007 (16.6)	<0.001	0.583	(0.535–0.635)	<0.001
Coronary heart disease, n (%)	1,797 (21.1)	1,105 (18.2)	<0.001	0.919	(0.845–1.034)	0.109
Heart failure, n (%)	347 (4.1)	174 (2.9)	<0.001	0.931	(0.799–1.881)	0.601
Renal Insufficiency, n (%)	839 (9.9)	477 (7.9)	<0.001	0.847	(0.754–0.966)	0.021
Peripheral artery disease, n (%)	347 (4.1)	286 (4.7)	0.063	-		
Anemia, n (%)	369 (4.3)	189 (3.1)	<0.001	0.836	(0.702–1.066)	0.104
Tumor, n (%)	603 (7.1)	386 (6.4)	0.088	-		
Deep vein thrombosis, n (%)	238 (2.8)	223 (3.7)	0.003	1.631	(1.365–1.950)	<0.001
SBP, mmHg, Mean ± SD	151 ± 24	148 ± 23	<0.001	0.998	(0.996–0.999)	0.006
DBP, mmHg, Mean ± SD	85 ± 15	85 ± 14	0.855	-		
Baseline NIHSS score, Median (IQR)^*^	6 (2–14)	3 (1–7)	<0.001	0.941	(0.934–0.946)	<0.001
Reperfusion therapy, n (%)	1,442 (17.0)	1,058 (17.5)	0.432	-		
Length of stay, days, Median (IQR)	10 (8–14)	11 (9–15)	<0.001	1.047	(1.042–1.053)	<0.001

*CI indicates confidence interval; DBP, diastolic blood pressure; IQR, interquartile range; NIHSS, national institutes of health stroke scale; OR, odds ratio; SD, standard deviation; SBP, systolic blood pressure; TIA, transient ischemic attack*.

* *NIHSS was available for 12,342 patients (5,324 in anticoagulant group and 7,018 in non-anticoagulant group)*.

### Factors Associated With Prior Use of Anticoagulation in Previously Diagnosed Patients With AF With a High Risk of Stroke

Among 7,807 patients with previously diagnosed AF and high risk of stroke (CHA_2_DS_2_-VASc ≥2), 1,585 patients (20.3%) had been receiving anticoagulation prior to the onset of stroke (17.9% anticoagulants only, and 2.4% were on both antiplatelets and anticoagulants). As seen in Table 4, patients who had been receiving anticoagulation had lower baseline NIHSS scores than those who are not. However, the mean international normalized ratio (INR) was 1.5 on the first test during hospitalization in patients receiving warfarin. Previously diagnosed patients with AF with a high risk of stroke who used anticoagulation were younger (OR 0.962, 95% CI 0.955–0.968), had a higher rate of ischemic stroke/TIA (OR 2.303, 95% CI 2.069–2.563), and peripheral artery disease (OR 1.456, 95% CI 1.176–1.795), and a lower rate of hemorrhagic stroke (OR 0.353, 95% CI 0.264–0.474) and hypertension (OR 0.634, 95% CI 0.562–0.717) compared with patients who do not use anticoagulants. Interestingly, patients who underwent anticoagulation before onset also had a higher rate of medication with antihypertensive, hypoglycemic agents, and statins.

**Table 4:** Univariate and multivariate analysis for prior use of anticoagulation in patients with previously diagnosed atrial fibrillation (AF) with high risk of stroke.

**Variable**	**Univariate analysis**			**Multivariate analysis**		
	**Non-anticoagulant group (n =6222)**	**Anticoagulant group (n =1585)**	***P* value**	**OR**	**(95% CI)**	***P* value**
Age, years, Mean ± SD	79 ± 8	76 ± 9	<0.001	0.962	(0.955–0.968)	<0.001
Male, n (%)	3,088 (49.6)	803 (50.7)	0.463	-		
**Prior medical history**						
Ischemic stroke/TIA, n (%)	2,213 (35.6)	847 (53.4)	<0.001	2.303	(2.069–2.563)	<0.001
Hemorrhagic stroke, n (%)	238 (3.8)	44 (2.8)	0.046	0.353	(0.264–0.474)	<0.001
Gastrointestinal bleeding, n (%)	134 (2.2)	36 (2.3)	0.775	-		
Urinary tract bleeding, n (%)	16 (0.3)	4 (0.3)	0.779	-		
Hypertension, n (%)	4,548 (73.1)	1,087 (68.6)	<0.001	0.634	(0.562–0.717)	<0.001
Diabetes, n (%)	998 (16.0)	288 (18.2)	0.041	1.020	(0.980–1.261)	0.336
Dyslipidemia, n (%)	68 (1.1)	25 (1.6)	0.113	-		
Coronary heart disease, n (%)	1,308 (21.0)	328 (20.7)	0.774	-		
Tumor, n (%)	408 (6.6)	89 (5.6)	0.170	-		
Renal Insufficiency, n (%)	248 (4.0)	73 (4.6)	0.267	-		
Peripheral artery disease, n (%)	315 (5.1)	114 (7.2)	0.001	1.456	(1.176–1.795)	0.003
Smoking, n (%)	1,331 (21.4)	345 (21.8)	0.746	-		
**Prior medication history**
Antiplatelets, n (%)	2,072 (33.3)	188 (11.9)	<0.001	0.103	(0.087–0.122)	<0.001
Antihypertensive agents, n (%)	3,121 (50.2)	955 (60.3)	<0.001	1.927	(1.714–2.164)	<0.001
Hypoglycemic agents, n (%)	678 (10.9)	227 (14.3)	<0.001	1.582	(1.177–2.071)	0.008
Statins, n (%)	1,113 (17.9)	459 (29.0)	<0.001	3.800	(3.271–4.406)	<0.001
**Clinical characteristics at stroke onset**						
SBP, mmHg, Mean ± SD	150 ± 23	145 ± 22	<0.001	-		
DBP, mmHg, Mean ± SD	85 ± 15	84 ± 14	0.004	-		
Baseline NIHSS score, median (IQR)^*^	5 (2–12)	4 (2–10)	<0.001	-		
CHA_2_DS_2_-VASc score, median (IQR)	4 (3–5)	4 (3–5)	<0.001	-		
Reperfusion therapy, n (%)	1,144 (18.4)	142 (9.0)	<0.001	-		
INR (on warfarin), Mean ± SD	1.1 ± 0.2	1.5 ± 0.6	<0.001	-		

*CI indicates confidence interval; DBP, diastolic blood pressure; IQR, interquartile range; INR, international standardized ratio; NIHSS, national institutes of health stroke scale; OR, odds ratio; SD, standard deviation; SBP, systolic blood pressure; TIA, transient ischemic attack*.

* *NIHSS was available for 6,654 patients (1,316 in anticoagulant group and 5,338 in non-anticoagulant group)*.

## Discussion

In this large prospective multicenter registry of stroke patients in China, <50% of patients with AIS with concurrent AF received guideline-recommended anticoagulation at discharge, which highlights a large care gap between guideline and practice. Prior hemorrhagic diseases and the presence of pneumonia during hospitalization were related to a lower rate of anticoagulant therapy. Furthermore, among our patients who were having a pre-index diagnosis of AF with a high risk of stroke and final ischemic event, only 20% received guideline-recommended OAC treatment, indicating the importance of AF management.

Guidelines from the European Society of Cardiology, European Heart Rhythm Association, and Heart and Stroke Foundation Canadian Stroke Best Practice Committees all highlight that weighing the risk of recurrence and bleeding is the core strategy to initiate anticoagulation after ischemic stroke (13–15). Several factors need to be considered in support of early or delayed anticoagulation. For example, early initiation of anticoagulation can be considered for young patients with lower NIHSS scores and well-controlled blood pressure, while delayed anticoagulation could be considered for ongoing intracerebral hemorrhage and gastrointestinal bleeding or combined renal insufficiency. Our study has similar findings.

Notably, we found several factors that are not recommended in the guidelines, which may explain the low proportion of anticoagulation in the real world. Prior hemorrhagic diseases, including intracerebral and gastrointestinal hemorrhage, were related to the absence of anticoagulants in this study. Increased undertreatment among patients with past bleeding events has been shown in previous studies (16, 17), indicating an excessive concern about the bleeding risk of patients. Waldo et al. found that perceived or actual bleeding risk was a significant predictor for warfarin undertreatment (18). Patient and family education on the benefits and risks associated with using anticoagulants should be made available and widely adopted by health care professionals. In the FibStroke study done in Finland, Palomäki et al. also found that patients with high HAS-BLED scores (HAS-BLED ≥3) were at an increased risk of OAC undertreatment than those with HAS-BLED <3 (17). Especially in the Asian population, a high rate of non-adherence to guidelines was found (6, 8, 9), which may be due to the higher rate of major bleeding, including intracranial hemorrhage in Asians than Caucasians (19–21). However, despite the fear of OAC-related bleeding complications in Asian patients, individualized assessment of bleeding risk is still needed (22, 23). Moreover, a high bleeding risk itself should not inevitably result in the decision not to use anticoagulants. Stroke and bleeding risk factors overlap, and patients at high risk of bleeding often have a high risk of ischemic stroke (24). To prevent bleeding while on treatment with anticoagulants, dynamic risk assessment to minimize the modifiable risk factors is of great importance (25).

The recommended general approach on the target timing of initiation of anticoagulation after stroke is as follows: 1 day or the same day after a TIA, 3 days after a mild stroke, 6 days after a moderate stroke, and 12–14 days after a severe stroke (26). In our study, the length of hospital stay was about 10 days in the non-anticoagulant therapy group without systemic bleeding during hospitalization, with their baseline NIHSS score only at 6 (indicating a mild-to-moderate stroke). It was also found that longer hospital stay was associated with increased odds of receiving anticoagulation. Therefore, these results revealed that there was a significant delay in the initiation of anticoagulation in the current clinical practice, which needs to be improved, potentially, by the education of clinicians.

Indeed, an increasing number of studies are focusing on the early prevention of hospitalized patients with AIS with concurrent AF. Data from observational studies and randomized controlled trials suggest that early recurrence of AIS in patients with concurrent AF ranges from 0.5 to 1.3% per day during the first two weeks (27, 28). In addition, early initiation of anticoagulation is particularly important for those who have a high risk of recurrences (29–31), such as those with high NIHSS scores and those with symptoms of atrial enlargement and atrial thrombus. Nowadays, small randomized trials (32) have reported that early initiation (1–5 days) of OAC treatment in patients with mild-to-moderate stroke or small-to-medium sized infarcts (less than a third of the affected arterial territory) could lead to both low frequency of symptomatic and asymptomatic intracranial hemorrhage and low rate of recurrent ischemic stroke. Furthermore, four randomized controlled trials [ELAN (NCT03148457; Switzerland), OPTIMAS (EudraCT, 2018-003859-38; UK), TIMING (NCT02961348; Sweden), and START (NCT03021928; USA)] which plan to recruit ~9,000 participants are underway, with methods that either use safer DOACs to initiate anticoagulation earlier or selecting the initiation time of OAC based on the risk judgment from the severity or imaging features of stroke (33). The results of these trials will help to persuade the clinicians to initiate early anticoagulation, improving the secondary prevention of stroke.

Guidelines recommend OAC for all patients with a high risk of stroke (CHA_2_DS_2_-VASc ≥ 2) unless contraindicated (34, 35). However, in this study, we found that only 20.3% of patients with AF with a high risk of stroke used anticoagulants prior to the onset of stroke, far lower than the current global anticoagulation rate of 42–60% (4, 36–38). The characteristics of patients receiving OAC before the index stroke include young age, fewer history of hypertension, and intracerebral hemorrhage. This phenomenon still reflects the excessive fear of anticoagulation-related bleeding in old patients and prior hemorrhage. It is worth noting that patients with higher compliance of medication for risk factors were more likely to accept anticoagulants, highlighting the importance of patient education for primary prevention.

For patients with AF taking warfarin, careful dosing and consistent INR monitoring are important. This is due to how warfarin efficacy depends on therapeutic INR control (INR range 2.0–3.0; if the presence of mechanical valve range is 2.5–3.5) and declines when the INR falls lower than 2.0 (14). It is also important to note that the mean INR in patients with prior warfarin use was only 1.5, which does not meet the targets, which could be a direct cause of the current stroke. Moreover, INR also influences the decision of acute treatment, as the rate of reperfusion therapy was significantly decreased in patients using anticoagulants prior to the stroke. In view of this, DOACs may be a better choice as treatment compared to warfarin. Overall, optimizing the anticoagulant strategy could be another direction to improve AF management in the future.

Notably, the presence of pneumonia during hospitalization was associated with reduced use of anticoagulants at discharge. This result is hard to explain as no research has shown that pneumonia increases the risk of anticoagulation. Stroke-associated pneumonia (SAP) was reported to be associated with poor prognosis (39), preventing clinicians from initiating anticoagulation in patients. It is unclear whether prevention and improved management of SAP for patients with AIS could increase the use of anticoagulants during hospitalization. Strengthening the prevention and management of complications during the acute phase may lead to improving the anticoagulation rate.

### Strength and Limitations

As large cohort research that used patient data from a prospectively constructed database with predefined variables, this study is prone to bias despite how the comprehensive data collection allowed us to take a closer look at specific comorbidities, such as systemic hemorrhage, anemia, and tumor. Another limitation was the lack of information on stroke etiology, size of stroke lesions, and outcomes of patients as data were not available in the complete database. Also, we included data from regional stroke units and both tertiary and secondary hospitals, indicating that our findings could be generalizable. Since patient records were documented by the treating physician, contraindications were sought out as thoroughly as possible in patients who did not receive anticoagulants. However, 11.5% of patients in this study were unclear for the deferring reason, which may underestimate the proportion of anticoagulation contraindications. Finally, the present study was a cross-sectional study. Hence, there is a need for further longitudinal studies to determine the differences made in survival/mortality or stroke recurrence due to undertreatment of AF. Despite these limitations, our study presents a realistic view of the situation where a clinician is placed when deciding whether to use anticoagulant therapy in a given patient. In addition, provides new directions for research and quality improvement targeting anticoagulation.

## Conclusions

Less than half of patients with AIS and AF received guideline-recommended anticoagulation at discharge, while only one in five patients with AF with a high risk of stroke had been using anticoagulants prior to the onset of stroke, which highlights a large care gap in hospitalized patients with stroke. To improve AF management in China, greater efforts must be made to educate both clinicians and patients to increase the rate of anticoagulation and optimize the anticoagulant strategy, including strengthening the health education of patients, improving the comprehensive acute stroke care quality, and enhancing the anticoagulant treatment experience of clinicians.

## Data Availability Statement

The original contributions presented in the study are included in the article/supplementary materials, further inquiries can be directed to the corresponding author.

## Ethics Statement

Written informed consent was not obtained from the individual(s) for the publication of any potentially identifiable images or data included in this article.

## Author Contributions

XG and HC performed the drafting and critical revision of the article and tables. ML had full access to all the data in the study, takes responsibility for the integrity of the data and the accuracy of the data analysis, performed conceptualization, drafting, and critical revision of the article. The remaining authors were involved in the critical revision of the article. All authors contributed to the article and approved the submitted version.

## Funding

This study was supported by the National Natural Science Foundation of China (81971101, 82171276) and the Science Technology Department of Zhejiang Province (2018C04011).

## Conflict of Interest

The authors declare that the research was conducted in the absence of any commercial or financial relationships that could be construed as a potential conflict of interest.

## Publisher's Note

All claims expressed in this article are solely those of the authors and do not necessarily represent those of their affiliated organizations, or those of the publisher, the editors and the reviewers. Any product that may be evaluated in this article, or claim that may be made by its manufacturer, is not guaranteed or endorsed by the publisher.
